# Implementation of a Standardized Initial Assessment for Demand Management in Outpatient Emergency Care in Germany: Early Qualitative Process Evaluation

**DOI:** 10.2196/18456

**Published:** 2020-09-04

**Authors:** Catharina Roth, Amanda Breckner, Jan Paulus, Michel Wensing

**Affiliations:** 1 Department of General Practice and Health Services Research University Hospital Heidelberg Heidelberg Germany

**Keywords:** emergency medical services, outpatient emergency care service, software, point-of-care systems, Germany

## Abstract

**Background:**

Inadequate assessment of the severity and urgency of medical problems is one of the factors contributing to unnecessary emergency department (ED) visits. The implementation of a software-based instrument for standardized initial assessment—Standardisierte medizinische Ersteinschätzung in Deutschland (SmED) (*Standardized medical Initial Assessment in Germany* in English)—aims to support health care professionals and steer patients toward the right health care provider. This study aimed to explore the implementation process of SmED from a user perspective.

**Objective:**

This study aims to evaluate the overall perception of SmED by health care professionals using the software, to examine to what extent SmED influences the workload and work routines of health care professionals, and to determine which factors are associated with the use of SmED.

**Methods:**

An early qualitative process evaluation on the basis of interviews was carried out alongside the implementation of SmED in 26 outpatient emergency care services within 11 federal states in Germany. Participants were 30 health care professionals who work with SmED either at the joint central contact points of the outpatient emergency care service and the EDs of hospitals (ie, the Joint Counter; *Gemeinsamer Tresen* in German) or at the initial telephone contact points of the outpatient emergency care service (phone number 116117). Matrix-based framework analysis was applied to analyze the interview data.

**Results:**

Health care professionals perceived that workload increased initially, due to additional time needed per patient. When using SmED more frequently and over a longer time period, its use became more routine and the time needed per call, per patient, decreased. SmED was perceived to support decision making regarding urgency for medical treatment, but not all types of patients were eligible. Technical problems, lack of integration with other software, and lack of practicability during peak times affected the implementation of SmED.

**Conclusions:**

Initial experiences with SmED were positive, in general, but also highlighted organizational issues that need to be addressed to enhance sustainability.

**Trial Registration:**

German Clinical Trials Register DRKS00017014; https://www.drks.de/drks_web/navigate.do?navigationId=trial.HTML&TRIAL_ID=DRKS00017014

## Introduction

In recent years, steadily increasing utilization of emergency departments (EDs) has aroused public and political attention, not only in Germany but in many nations [1-7]. Different studies have examined factors that contribute to increased numbers of emergency admissions [5,8,9]. Factors that may explain high use of emergency resources include an aging population, increased number of chronically ill people, lack of cost awareness, lack of accessibility, unclear organization of outpatient emergency care service, and patients’ personal assessments regarding severity and urgency of medical conditions [9-11]. The high use of EDs negatively affects not only patients (eg, long waiting times, reduced patient satisfaction, and higher mortality) but also health care professionals (eg, high workload, work-related stress, safety, and efficiency issues) working at the ED [2,4,9,12]. A range of strategies have been applied to address this issue by policy makers [6].

One category of strategies are programs and policies that aim to reduce the number of unnecessary ED visits. Unnecessary ED visits are defined as using the ED for health issues that do not require immediate medical treatment. Those visits could be classified as *inappropriate*, because ED resources are used for health issues that can be treated elsewhere (eg, in primary medical care or outpatient emergency care) [13]. The estimated prevalence of inappropriate visits at EDs is between 20% and 40% [13,14]. Factors associated with inappropriate use of EDs include patient age, education, and absence of family support [14]. In addition, inadequate assessment of severity and urgency of presented health problems determine inappropriate ED visits [4,14,15].

**Figure 1:**
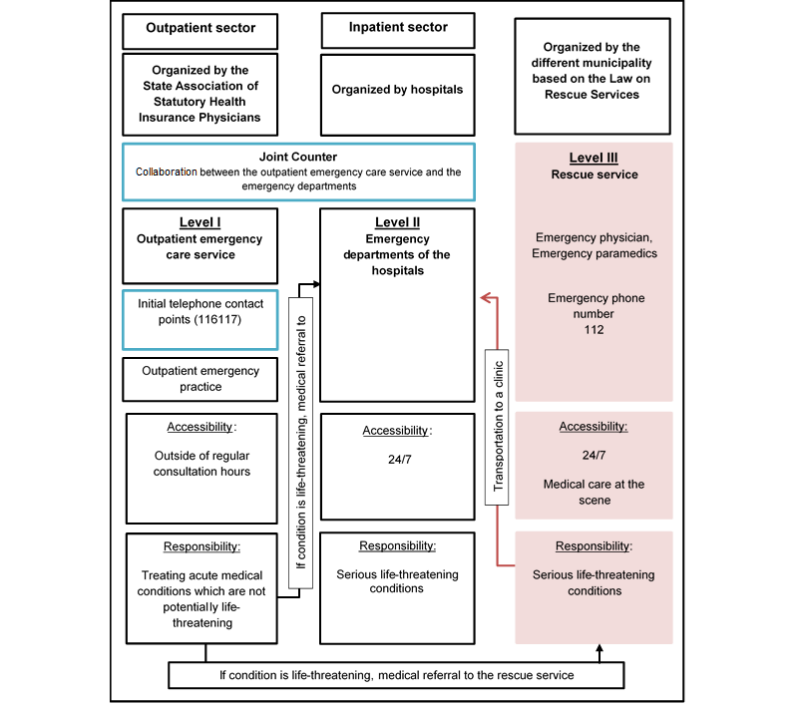
Overview of the three different levels of emergency care in Germany.

In Germany, the organization of emergency care service is complex [9]. The National Association of Statutory Health Insurance Physicians distinguish between three levels of emergency care services: the outpatient emergency care service (Level I), the emergency care service of hospitals (Level II), and the rescue services (Level III) [16]. The outpatient emergency care service is for acute medical conditions that are not potentially life-threatening that are presented outside of regular consultation hours. This service can be subdivided into the initial telephone contact points (ie, phone number 116117) or outpatient emergency practices. Outpatient emergency practices can be regular primary care physician practices or can be located at a hospital. The phone number 116117 is responsible for steering patients with medical conditions toward the right point of care. Patients calling this number have different options depending on their urgency for medical treatment. They can be advised regarding self-treatment at home, receipt of medical treatment by the primary care sector within the next few days depending on regular consultation hours, medical referral to an outpatient emergency practice, home visit by an outpatient physician, medical referral to the ED, or medical referral to the rescue service, based on their urgency for medical treatment. The Joint Counter (*Gemeinsamer Tresen* in German) is a cooperative arrangement between the outpatient emergency care service (Level I) and the EDs of the hospitals (Level II). Patients who visit the Joint Counter are either admitted to the ED or treated by the outpatient emergency care service (see Figure 1) [16].

Since the beginning of 2019, a computer-based software—Standardisierte medizinische Ersteinschätzung in Deutschland (SmED) (*Standardized medical Initial Assessment in Germany* in English)—has been implemented within the Joint Counter and the phone number 116117 to support health care professionals to steer patients toward the right point of care and, therefore, reduce inappropriate ED visits: both settings are highlighted in blue in Figure 1. Previous research showed that many complex interventions (eg, implementing a computer-based software like SmED) that have been shown to be effective failed to be implemented sustainably and widely in health care practice [17,18]. Many types of factors can act as barriers or facilitators of implementation of changes, including individual, organizational, and health system–related factors [19,20]. It is crucial to understand these factors in order to optimize the speed and comprehensiveness of implementation [17]. This study aimed to answer the following research questions: (1) What are health professionals’ overall perceptions of SmED? (2) To what extent did SmED influence workload and work routines of health care professionals? and (3) Which factors were associated with the use of SmED?

## Methods

### Study Design, Ethics Approval, and Trial Registration

This early process evaluation aimed to investigate the following: Reach, Effectiveness and Efficacy, Adoption, Implementation, and Maintenance (RE-AIM), according to the RE-AIM framework [21]. A qualitative process evaluation on the basis of interviews with health care professionals was carried out alongside the implementation of SmED. Ethics approval was obtained from the Medical Ethics Committee of the Medical Faculty Heidelberg (S-640/2018) prior to the start of the study in October 2018. The study has been registered at the German Clinical Trial Register prior to the start of the study (registration No. DRKS00017014).

### Study Setting

The overall aim of the DEMAND project is to improve medical care of patients who present with an urgent need for emergency treatment and/or medical advice on the basis of a more efficient use of emergency care resources. The implementation of a software-based instrument for standardized initial assessment (ie, SmED) aims to support health care professionals (eg, nurses, physician assistants, and paramedics) in emergency care and to steer patients with nonurgent medical needs toward the right point of care. The State Associations of Statutory Health Insurance Physicians have implemented SmED since March 2019 at the Joint Counter, as well as the phone number 116117, in 11 of the 16 federal states of Germany. Health care professionals within this setting were expected to change their service delivery by using the software and, therefore, influence individuals in the target population, which included patients who contact the Joint Counter or call 116117 regarding the need for medical treatment and/or advice.

### Study Population

The Institute for Applied Quality Improvement and Research in Health Care (aQua Institute) invited all State Associations of Statutory Health Insurance Physicians from each federal state of Germany to take part in this project. Out of 16 federal states, 11 agreed to take part. No specific reasons for the nonparticipation of the other five federal states were identified. The State Associations of Statutory Health Insurance Physicians informed the research team at the University Hospital Heidelberg about all health care professionals using SmED and were, therefore, potential participants. According to this number, information packages, including an invitation letter, an information leaflet, and an informed consent form for tape recording, were put together and sent to a contact person responsible for distributing. The information sheet included contact details of research team members who were available to participants to discuss the study or address additional concerns or questions. Participants who decided to take part in an interview were requested to contact the researchers directly. An informed consent form for tape recording was signed prior to the start of the interview by the participant and the interviewer. Different strategies, including email reminders, telephone calls, and the annual project coordination team meeting, were used to maximize response rate.

### The Intervention

SmED is a computer-based software that requires an internet connection. The software can be used by health care professionals (eg, nurses, practice assistants, and paramedics) for initial assessment as a basis for demand management in outpatient emergency care services. The purpose of this software is to support health care professionals and to steer patients toward the right point of care based on their actual medical needs. SmED can be used in different outpatient emergency care services and applies a number of well-defined questions regarding different medical disorders and issues. SmED uses an algorithm based on the *red flag* approach to rate the urgency of patients. There are four different degrees of urgency: (I) emergency, (II) fastest possible medical treatment by a physician, (III) medical treatment by a primary care physician within 24 hours, and (VI) medical treatment within the next couple of days or telephone consulting. Patients are steered toward the right point of care based on this algorithm. In this project, SmED is used at the Joint Counter and during calls to the telephone number 116117. The software can be used immediately and at any time. However, in this project the sites decided individually when and how often they would use SmED. SmED was developed on the basis of an available system from Switzerland—the Swiss Medical Assessment System (SMASS)—and adapted to the German health care system before implementation. Regular software updates based on user feedback were performed to modify SmED during implementation.

### Implementation Activities

The aQua Institute organized workshops prior to the implementation of SmED for all State Associations of Statutory Health Insurance Physicians from each participating federal state of Germany. A training concept for potential users and trainers was designed. Additionally, a data protection concept and an implementation plan for each project site was developed. Moreover, quality management and a support management program responsible for implementation sustainability were introduced.

### Measures and Data Collection

Semistructured telephone interviews were conducted by the two first authors (AB and CR) with 30 health care professionals who used SmED between July and December 2019. The semistructured interview guideline (see Multimedia Appendix 1) was developed on the basis of the RE-AIM framework [22]. It covers the following themes: reach, effectiveness and efficacy, adoption and uptake, implementation, and maintenance and sustainability. These themes, as described in the original description of the RE-AIM framework [22], were translated into questions in the context of SmED. All interviews were audio recorded with the consent of all participants and ranged in length from 8 to 39 minutes. The interviews were pseudonymized and transcribed verbatim. Transcripts were not returned to participants for comment or correction. All interview quotes were translated into English by the first author (CR).

### Data Analysis

The matrix-based method of framework analysis according to Ritchie and Spencer [23] and Gale et al [24] was conducted. This analysis is seen as an appropriate content analysis approach in a study where a conceptual framework is available at the start of the study [24]. As a first step, all interviews were transcribed verbatim (*Step 1: Transcription).* Additionally, all transcripts were reviewed by the two interviewers (AB and CR) for accuracy. After verbatim transcription of the interviews, the two first authors (AB and CR) became familiar with the whole dataset (*Step 2: Familiarization*). In step three, the first two interviews were deductively coded independently by the two first authors (AB and CR). Codes and themes of interest were defined based on the interview guide and on the RE-AIM framework. A few themes of interest were identified inductively from the data during the analysis (*Step 3: Coding*). The results were discussed and a final coding system based on the RE-AIM framework was developed for further analyses (*Step 4: Developing a working analytical framework*). The remaining interview transcripts were assigned to the existing codes by one researcher (CR or AB) and checked by a second researcher (AB or CR) (*Step 5: Applying the analytical framework*) [24]. The final step was to analyze whether there were any differences or connections between the codes. Interview data were analyzed using MAXQDA, version 2018.1.0, a computer-assisted qualitative data management software. Additionally, participant characteristics were analyzed descriptively using SPSS Statistics for Windows, version 25.0 (IBM Corp).

### Availability of Data and Material

The dataset generated and analyzed during this study will not be made publicly available due to European Data Protection Law but may be available by the corresponding author upon reasonable request.

## Results

### Overview

At the beginning of the study, according to the project coordinator, 391 health care professionals used SmED. All 391 professionals were invited to take part in an interview and 30 decided to take part. Characteristics of the participants are described in Table 1.

**Table 1:** Characteristics of health care professionals.

Characteristic		Value (N=30)
**Gender, n (%)**		
	Female	23 (77)
	Male	7 (23)
Age (years), mean (SD)		43.3 (10.1)
**Federal state of Germany, n (%)**		
	Baden-Wuerttemberg	1 (3)
	Bavaria	3 (10)
	Berlin	3 (10)
	Bremen	6 (20)
	Hesse	4 (13)
	North-Rhine	2 (7)
	Rhineland-Pfalz	5 (17)
	Schleswig-Holstein	2 (7)
	Thuringia	1 (3)
	Westphalia-Lippe	3 (10)
**Professional qualification, n (%)**		
	State-qualified nurse	3 (10)
	Practice assistant	17 (57)
	Emergency paramedic	7 (23)
	Controller or dispatcher	1 (5)
	Other	2 (7)
Work experience (years), mean (SD)		20.37 (9.62)
**Period of time using SmED^a^, n (%)**		
	1 month	1 (3)
	Between 1 and 2 months	1 (3)
	Between 2 and 3 months	4 (13)
	Between 3 and 4 months	4 (13)
	Between 4 and 5 months	1 (3)
	Between 5 and 6 months	5 (17)
	Over 6 months	14 (47)
**Setting, n (%)**		
	Initial telephone contact point	18 (60)
	Joint Counter	10 (33)
	Both settings	2 (7)

^a^SmED: Standardisierte medizinische Ersteinschätzung in Deutschland (*Standardized medical Initial Assessment in Germany* in English).

### Theme 1: Reach

Regarding the target population of SmED, the health care professionals explained that SmED is not applicable to patients with hearing impairment (1/30, 3%); elderly patients (2/30, 7%); terminally ill patients (2/30, 7%); patients who are incapable of answering questions due to, for example, neurological diseases (3/30, 10%); or patients suffering from alcohol or drug overdose (1/30, 3%). SmED is also not feasible for patients where someone else (eg, husband or wife) is calling on their behalf (3/30, 10%), patients with psychiatric disorders (5/30, 17%), or patients with language barriers (14/30, 47%). Moreover, SmED is not applicable if qualified health care professionals (eg, nurses working at a nursing home) call on the behalf of a patient (8/30, 27%). Health care professionals working at the initial telephone contact point (ie, phone number 116117) also reported that they do not use SmED for patients who want to order a prescription or for those seeking simple advice (3/30, 10%). Health care professionals working at the Joint Counter explained that, furthermore, SmED is not feasible for patients who are regular visitors to a long-term therapy service (1/30, 3%) or who are in poor medical condition (6/30, 20%). One health care professional stated that patients with chronic diseases or nursing diagnoses (eg, having a urinary catheter), for example, are not included.

80-year-old Turkish person who speaks very little German does not understand the questions and then there is no one to translate, and then SmED, it serves no purpose.

Participant working at the Joint Counter

### Theme 2: Effectiveness and Efficacy

This theme concerns the impact of SmED on steering patients toward the right point of care and the impact of SmED on workload and working methods. A vast majority of health care professionals (21/30, 70%) stated that SmED rates the urgency for medical treatment higher than they themselves would rate the urgency. For instance, if patients report high blood pressure (4/30, 13%), infection of the gastrointestinal system (2/30, 7%), respiratory problems (3/30, 10%), fever and chills (3/30, 10%), severe pain (3/30, 10%), or uncontrolled falls (1/30, 3%), SmED immediately rates them as emergencies. Almost two-thirds of all participants (18/30, 60%) reported that if SmED rates the urgency for medical treatment higher than they expected, they changed the decision based on their professional experience or after consultation with a physician. If the health care professionals changed the category as rated by SmED, they documented and justified it on the final summary. Only 5 participants out of 30 (17%) said that they had to accept how SmED rated patients even though they disagreed with the software.

Handling of patients who are rated as nonurgent varied, depending on the setting. Patients who call the phone number 116117 and are classified as nonurgent are advised to visit an outpatient emergency practice (4/30, 13%), are forwarded to telephone counseling with a physician (9/30, 30%), receive a home visit by the emergency care physician (5/30, 17%), or are given advice (eg, going to the primary care physician within the next few days or treating themselves at home) (6/30, 20%). At some initial telephone contact points, patients are given the opportunity to call back if their medical condition is getting worse (3/30, 10%). Patients who contact the Joint Counter are treated by a physician within the next few hours but have to wait (9/30, 30%) or are referred to the outpatient emergency care sector (1/30, 3%). Due to legal requirements, all patients who contact the Joint Counter have to be treated or seen by a physician; thus, at three Joint Counters, waiting lists based on SmED categories are implemented to organize patients.

### Theme 3: Adoption and Uptake

This theme concerns whether health care professionals are willing to use SmED. When asked about expectations or disappointments, one-third of the participants said they expected SmED to be an aid for decision making, especially for unexperienced colleagues (11/30, 37%). However, health care professionals stated that their professional experience influenced decisions concerning urgency of medical treatment independently of the software. In addition, their intuition, professional knowledge, and experience influenced how or if they used the software. Health care professionals stated that after working in the field of emergency care service for many years they are able to make safe decisions and rate the urgency for medical treatment of patients without a support system (18/30, 60%).

Well, meanwhile, I personally would say that especially for younger coworkers, it is a chance to ask structured questions. And it does not matter from where the patient is calling, we ask the same questions and eventually it is always the same result...

Participant from telephone contact point (phone number 116117)

Health care professionals stated that they assumed that SmED could function as a tool for quality assurance (eg, as a standard tool) (4/30, 13%). According to 7 out of 30 (23%) health care professionals, SmED provides support regarding decision making and helps to structure the assessment and, therefore, to correctly assess patients’ needs for medical treatment. Nevertheless, one-third (11/30, 37%) of the participants reported that the tool is imprecise and unstructured. The order of the questions sometimes does not match a patient’s symptoms. One advantage according to the health care professionals is that they do not forget questions and the system provides an opportunity for a structured assessment (8/30, 27%). In addition, questions asked after implementation have not been asked before (eg, whether the patient has been abroad lately) (1/30, 3%). Nevertheless, 4 health care professionals out of 30 (13%) mentioned that it is possible to skip questions if they are unnecessary or inappropriate, based on their professional judgment.

I think the main advantage is that the conversation is structured...And yes, that we identify people who dramatically report their symptoms even though they aren’t that urgent. Yes, I think that we identify them.

Participant from telephone contact point (phone number 116117)

Although health care professionals expected SmED to be more patient oriented (8/30, 27%), some stated that it includes too many questions (3/30, 10%). Particular questions regarding medication or drugs are too complicated to answer for some patients. A small number of participants said SmED is too comprehensive (6/30, 20%). Moreover, almost one-third of interviewees (8/30, 27%) explained that they have the perception that a small group of patients are being rude and are annoyed due to busy lines, long waiting times at the Joint Counter, or being asked too many questions.

...the conversation definitely takes longer and, therefore, the lines are busy. Patients are sometimes annoyed if they have to wait 15 to 20 minutes instead of 10 minutes due to the additional time needed per call. Sometimes I have to ask comprehension questions like “What do you mean by that?” and that’s definitely a disadvantage.

Participant from telephone contact point (phone number 116117)

In addition, if a patient is feeling really sick or if the situation is too challenging, answering all SmED questions puts an additional burden on the patient (2/30, 7%). Furthermore, if a patient is calling 116117 and the lines are busy, they may decide to hang up and call the rescue service.

I always think that if someone is really feeling sick, SmED might be a burden. I mean, I do not have feedback regarding that, no one told me that, but I have the feeling that SmED is too much for some patients.

Participant from telephone contact point (phone number 116117)

However, when asked about SmED’s impact on patients, almost half of the participants (14/30, 47%) explained that they have the perception that patients, in general, feel like they are in good hands and that they are grateful for a more structured and comprehensive assessment. Thus, according to 6 professionals out of 30 (20%), using SmED increases patient safety.

...and a lot of patients like that we have to talk with them a little longer, they are like “It was very kind of you that you took that much time for me.”

Participant from telephone contact point (phone number 116117)

The majority of health care professionals reported that one barrier was that using SmED increases their workload due to the additional time needed per call, per patient (21/30, 70%). However, through more frequent use of SmED, it became more routine and the additional time needed per call, per patient, decreased (9/30, 30%). A facilitator for implementation was motivation through colleagues, trainers, or leaders (3/30, 10%). If these persons are convinced by SmED, they were able to encourage other health care professionals. On the other hand, health care professionals who see only the negative effects of the new software can have a negative impact on successful implementation of SmED.

First of all, long-term employees have to be convinced because they are used to the old system. And yes, during work life, people are usually confronted with new things; however, Germans tend to only see the negative sides of new things at the beginning.

Participant from telephone contact point (phone number 116117)

### Theme 4: Implementation

This theme concerns the consistency of the organization and adjustments made during and prior to the delivery of the intervention as well as implementation strategies. All interviewees (30/30, 100%) reported that they participated in a training session prior to implementation. The development and structure of SmED and its beginning in Switzerland were presented. During the training session, participants worked with exemplary patient cases. The success of the training sessions was monitored differently. However, nearly one-third of all health care professionals (8/30, 27%) stated that they used patient cases to learn how to use SmED. Those cases were discussed at the end of the training session. Out of 30 participants, 1 (3%) said that they used SmED once during their working hours with different patient cases to understand how the software works in real-life situations. Another participant (1/30, 3%) explained that they had to use SmED five times during their working hours and documented it. Out of 30 participants, 1 (3%) explained that they watched web-based videos and tested their knowledge afterward. A total of 5 out of 30 (17%) interviewees stated that they were able to ask experienced colleagues further questions at the start of the implementation of SmED to learn how to use the software correctly. The overall training was evaluated as completely positive by 20 out of 30 (67%) interviewees. Out of 30 participants, 1 (3%) brought up that it was too much input for one day. A total of 5 out of 30 (17%) participants mentioned that the theoretical part was too long and 4 (13%) said that more practical examples would have been beneficial.

We had a training session and after that SmED was installed by Medistar, which is the software provider. We then could use the test version to get familiar.

Participant working at the Joint Counter

Almost all participants (26/30, 87%) reported that they had not used an initial-assessment software prior to implementation of SmED. Health care professionals explained that they had internal standards and a guideline for conversations with patients but could ask questions individually. A total of 4 health care professionals out of 30 (13%) stated that they had access to another initial-assessment software, which prioritized and categorized patients.

Prior to the start of using SmED, 3 participants out of 30 (10%) from the Joint Counter stated that they introduced a system where patients first pull a number and are then transferred to a waiting area further away from the counter due to data protection law. Out of 30 interviewees, 2 (7%) mentioned that they increased their number of employees.

### Theme 5: Maintenance

This theme concerns the extent to which SmED becomes institutionalized or part of the routine organizational practices and policies. Different facilitators and barriers influencing implementation of SmED were identified by the health care professionals. One barrier according to the interviewees (10/30, 33%) is that currently an integration with other software is not possible. Participants explained that a closer connection of SmED with the information system used in daily practice would increase acceptance and feasibility. At this time, health care professionals stated that they had to work with both software programs in parallel. This had a massive impact on workload. Implementing an interface between the two software programs would, therefore, enhance implementation sustainability.

One problem is that there is no link or interface between SmED and the software usually used.

Participant from telephone contact point (phone number 116117)

Nevertheless, I think if there is a link between SmED and our software, acceptance by the employees will increase and, with a higher acceptance, I will get more experienced and have a better understanding.

Participant from telephone contact point (phone number 116117)

Health care professionals stated that they were disappointed due to challenging technical problems they face during their daily working routines. Hence, SmED is not always practicable (5/30, 17%) due to, for example, a poor internet connection. A total of 2 professionals out of 30 (7%) said that SmED is a computer-based program that is error prone (2/30, 7%) and can be tricked; for example, patients who are frequent callers know how to answer in order to be rated as urgent (1/30, 3%). A total of 2 health care professionals out of 30 (7%) explained that another barrier for successful implementation could be lack of employees in the future. At this time, additional time needed per call, per patient, already increases the workload per employee.

We will definitely need more employees; this is crucial, since the number of incoming calls has increased gradually and also time needed per call rose due to using SmED...

Participant working at the Joint Counter

Moreover, using the software during peak times or at times with higher call volumes, such as during national holidays, is not feasible. Almost one-third of participants (9/30, 30%) reported that a large volume of incoming calls during the use of SmED increases pressure on employees and, thus, induces work-related stress. Health care professionals working at the Joint Counter stated that SmED is not practicable if the number of patients waiting is high (6/30, 20%).

At the moment, we use SmED only three days per week: Monday, Tuesday, and Thursday. I guess we are also supposed to use it on weekends and national holidays sometime soon. I think it is not feasible to use it then, due to the high number of patients visiting us.

Participant working at the Joint Counter

Another barrier described by 1 participant (3%) is that sharing information with the next point of care is currently not possible. On the other hand, 1 health care professional (3%) mentioned that information can be shared between the Joint Counter and the rescue service. Thus, patients calling 112—the emergency phone number—inappropriately can be steered to the right point of care easily. A total of 4 (13%) participants from the Joint Counter described problems regarding data security and privacy. According to the health care professionals, separate rooms are needed to use SmED to prevent invasion of privacy. At this time, patients usually wait in a queue and can hear what the person in front of them is being asked.

...the problem is data protection! Patients sit here or stand here and then we ask them questions and another person is standing behind them. Even though we say, “Could you keep a proper distance please...”

Participant working at the Joint Counter

A total of 2 (3%) health care professionals reported that, although they use SmED, a physician could be sitting next to them asking the patient the same questions and could rate the urgency based on her or his experiences and not based on SmED results. Nearly half of all health care professionals (14/30, 47%) described that, often, physicians do not read the summaries created by SmED. According to 11 participants (37%), summaries were only partly read. Thus, questions may be asked twice. This perhaps could give patients the impression that the professionals do not communicate effectively. Moreover, the health care professionals using SmED feel like their work is unnecessary.

Impact on the patient? I think...in general...patients like being questioned at first...it is not disadvantageous. However, sometimes the physician asks the same questions because they do not read the summary and then the patient thinks we do not communicate...

Participant working at the Joint Counter

A total of 1 (3%) interviewee stated that developing a simpler version of SmED that can be used at the Joint Counter while examining patients may positively influence implementation sustainability. Moreover, regular software updates including user feedback (eg, symptoms that are missing) were needed during implementation. More than one-third of all participants (11/30, 37%) described that they could collect suggestions for improvement and share this information with software engineers. If appropriate, those suggestions could be integrated into SmED within the next software update, which could facilitate implementation. A total of 2 (7%) participants stated that this will support implementation sustainability. Additional training will also improve maintenance. Another facilitator for maintenance mentioned by 4 (13%) interviewees is that SmED is a medical product and, therefore, assures legal certainty. The *red flag system* or the *priority list* (4/30, 13%), which helps to easily identify emergency patients, is another benefit.

More than half of the participants (16/30, 53%) explained that the medical responsibility lies with the health care professionals answering the call or admitting the patient to the ED. However, depending on the software ranking, responsibility stays with the professionals or is handed over to the next point of care. The other half (12/30, 40%) reported that the medical responsibility always lies with a physician. A total of 1 (3%) participant stated that after implementing SmED, liability is placed on the medical product, which enhances implementation sustainability. According to 4 (13%) participants, there are neither facilitators nor barriers influencing the implementation, since it will be binding for all State Associations of Statutory Health Insurance Physicians to use SmED for initial assessment from 2020 onward.

Our expectations have been met. We are legally protected, we do not forget to ask certain questions, and it is a support for decision making. It is perfect for us.

Participant from telephone contact point (phone number 116117) and the Joint Counter

## Discussion

### Principal Findings

This study focused on the perceptions of health care professionals at an early stage of the implementation of SmED. In general, health care professionals evaluated SmED positively. Workload increased initially, due to additional time needed per call, per patient. If SmED had been used more frequently and over a longer time period, its use by health care professionals would have become more routine, which would have a positive impact on time needed per call, per patient. SmED was perceived to support decision making regarding urgency for medical treatment, albeit not all patients were eligible. Technical problems, lack of integration with other software, and lack of practicability during peak times influenced the implementation process negatively. Eliminating given barriers may influence uptake and implementation sustainability.

### Comparison With Previous Work

#### Reach

SmED is not applicable to all patients, neither at the Joint Counter or at the initial telephone contact points. This group seems small, but includes patients with complex needs who may frequently contact out-of-hours care. Particularly, patients with neurological diseases [25] or psychiatric disorders [26] need to be steered toward the right point of care based on their urgency due to the risk of sudden or unexpected deterioration. The same is true for patients with alcohol intoxication [26]. According to a study by Pajonk et al [27], the number of psychiatric emergencies has increased in recent decades. It, therefore, seems problematic that SmED does not depict those cases.

#### Effectiveness and Efficacy

The overall aim of SmED is to improve emergency care for patients with urgent conditions, steer patients with nonurgent medical needs toward the right point of care, and, therefore, disburden the EDs. The urgency for medical treatment rated by SmED was perceived to be higher than health care professionals’ assessments. This may have an impact on the effectiveness of SmED to identify patients with nonurgent conditions and to steer them toward the right point of care. This finding is consistent with those by Jansen Van Eijndt et al [28]. They had more urgent patients after implementing a computer-based triage system. A more defensive algorithm identifying nonurgent patients may be needed to improve the effectiveness of SmED regarding steering nonurgent patients toward the right point of care. A systematic review by Huibers et al [29] concluded that, on average, patient safety is high if patients with nonurgent medical symptoms contact the out-of-hours service via telephone. However, there is room for enhancing patient safety for patients who present with highly urgent medical symptoms [29]. They found that an average of 10% of telephone triage contacts with patients reporting urgent symptoms resulted in different adverse events, such as medical errors or unplanned ED attendance. In our study, health care professionals stated that patient safety, in general, increased after implementation of SmED. This finding agrees with the results of a study by Van Ierland et al [30] evaluating the validity of the Netherlands Triage System (NTS). They detected an increasing tendency toward more ED referrals among high-urgency-level patients and an increased amount of self-care advice among lower-urgency-level patients for the computer-based telephone triage. The urgency levels ranked by the systems matched with the majority of those using the system; thus, NTS seems to be feasible. However, an independent standard identifying true undertriage and overtriage may be needed [30]. According to Scherer et al [9], the main reason for overcrowding EDs is patients who do not require urgent medical care. Hence, the objective to address this problem may not be achieved, due to lack of effectiveness described above. In addition, at the Joint Counter, patients rated as nonurgent are treated by a physician but have long waiting times, which contribute to the overcrowding. Nevertheless, due to legal regulations (§ 27 SGB V), employees of the Joint Counter are ordered to treat every patient. Patients rated as nonurgent at the initial telephone contact points (ie, phone number 116117) are admitted to the outpatient emergency practice, connected to telephone counseling, or given telephone advice. However, at this point in time it is not clear whether patients accepted this procedure or whether they still sought medical treatment at the ED after calling 116117.

Health care professionals stated that their workload increased due to more time needed per call, per patient, when using SmED. This has a negative impact on the efficacy of the professionals. This finding is supported by the results of Porter et al [31]. In their study, they evaluated the experiences of paramedics using a computerized clinical decision support (CCDS) system. The interviewed paramedics reported that they need more time at the scene when using the CCDS system. Nevertheless, this finding disagrees with those by Ong et al [32]. In their study, they investigated whether there is a difference in call duration and triage decisions in out-of-hours cooperatives when using or not using an expert system. Professionals interviewed in our study stated that their perceptions regarding additional time needed changed positively over time due to frequent use of SmED, which enhances routine and, in turn, has a positive impact on efficacy.

#### Adoption

SmED can function as a decision-making process regarding urgency for medical treatment support for unexperienced professionals. Nevertheless, many years of professional experience and knowledge were perceived as a basis for safe decision making. These findings correspond with those by Snooks et al [7], who investigated paper-based protocols to assess and triage patients, regarding their need for transportation to the ED. According to the findings by Snooks et al [7], decisions were influenced mainly by experience. Porter et al [31] concluded that paramedics would rather trust their clinical skills than the software [31]. Chang et al [33] described that professional experience and intuition plays an important role for nurses while using a triage system and improves decision making; these findings are consistent with those by Smits et al [34], Van der Linden et al [35], and O’Cathain et al [36]. Smits et al [34] investigated telephone triage in general practices in the Netherlands and concluded that triage accuracy is higher among practice assistants with more years of professional experience. Van der Linden et al [35] investigated whether ED crowding affects triage processes in the Netherlands. They found that more patients were redirected to general practice care when the nurses were more experienced. O’Cathain et al [36] evaluated whether different types of nurses differ regarding their triage decisions. They observed that length of clinical experience and type of software used influenced decision making. Nurses with more experience were more likely to give advice regarding self-treatment at home compared to less experienced nurses [36]. Nurses with more years of experience have more professional knowledge and clinical skills compared to professionals with fewer years of experience; therefore, decision making differs [37]. Although SmED supports decision making among younger professionals, professional experience is perceived as an important factor.

Health care professionals expected SmED to be a tool for legal protection and quality assurance. However, at this point in time it is not clear if SmED can provide legal certainty. Moreover, health care professionals are able to skip questions; therefore, the concept of using a software program as a nationwide standard for quality assurance may not be fulfilled. Porter et al [31] support this finding. According to their results, several paramedics used CCDS systems retrospectively to save time and not as a decision support. SmED uses an algorithm based on the *red flag* approach. It is not clear whether only relying on this algorithm is acceptable or if the experience of health care professionals regarding patient safety should be considered. Interestingly, the perception of the structure of SmED varied between imprecise and precise. The participants reported that they can skip questions and that the order of the questions is inappropriate. However, SmED provides a system for a structured initial assessment and functions as a reminder for crucial questions. These findings agree with those by Porter et al [31], who found that health professionals explained that CCDS systems reminded them to do all necessary checks. Patients’ satisfaction increased due to more structured and comprehensive assessments. This finding is supported by Snooks et al [7], where patients’ satisfaction with their care was higher due to more in-depth assessments.

#### Implementation and Maintenance

In general, the introduction of SmED and training prior to implementation was evaluated positively. However, implementation slightly differed among the project sites. Success of implementation was ensured differently, therefore, continuity was not given. This may have an impact on how health care providers evaluated and accepted SmED in general. The main barrier mentioned by the professionals is the lack of integration with the software that was used parallel to SmED. This finding is consistent with those of Porter et al [31], who detected a variety of technical problems (eg, difficulties keeping the PC charged or printing patient records). Health care professionals also assumed that SmED could be a tool for quality assurance but, contrary to expectations, SmED still has some technical limitations, such as a lack of practicability due to a poor internet connection. These results agree with those by Porter et al [31], where paramedics reported having problems with the CCDS system, since it is web based and, thus, not practicable without an internet connection.

According to regulation § 75 SGB V, the outpatient emergency care sector is the first contact point for patients outside of regular consultation hours (eg, during national holidays or on the weekend). However, use of SmED is not feasible during peak times or at times with higher call volumes (eg, national holidays and weekends). Using SmED will be binding for all State Associations of Statutory Health Insurance Physicians at the initial telephone contact points (ie, phone number 116117) [38]. In addition, advertisements for the phone number 116117 have increased, with more marketing planned in 2020. Thus, the number of patients calling 116117 may rise further. More employees may be needed to ensure that all calls can be answered within an appropriate time period. High incoming call volumes have already increased work-related pressure. Additional time needed per call may exacerbate this issue. Nevertheless, it is not clear whether SmED is the only related factor.

### Limitations and Strengths

This study was carried out in only 11 federal states of Germany; including the missing states could have identified new aspects. Although respondents were mostly outspoken about their perceptions, social desirability cannot be precluded. It is possible that comparability may not occur, due to the long time period for conducting the interviews. Moreover, health care professionals started to use SmED at different time points, which may have influenced their perceptions as well. Although different reminders to increase the response rate were used, only a low number of participants were reached. This may be due to not having contacted the health care professionals personally. This analysis was guided by an appropriate methodological strategy to minimize research bias and reduce the risk of losing relevant content. In addition, the analysis was done by the two first authors individually and was compared during several meetings to ensure consistent coding.

### Conclusions

This study is probably the first study to investigate perceptions of health care professionals regarding a computer-based instrument for standardized initial assessment in Germany. Despite the limitations, this study shows that using a software-based instrument for standardized initial assessment supports health care professionals’ decision making. Nevertheless, SmED rates patients’ urgencies as higher than do the professionals; it is, therefore, not clear whether SmED steers patients with nonurgent medical needs toward the right point of care, thereby disburdening the EDs. The findings of this study could help to implement SmED in additional federal states of Germany, as well as to implement similar computer-based initial-assessment software systems in other European countries.

## Appendix

Multimedia Appendix 1 Translated interview guide used to conduct the qualitative interviews with health care professionals.

## Abbreviations

aQua Institute: Institute for Applied Quality Improvement and Research in Health Care
CCDS: computerized clinical decision support
ED: emergency department
NTS: Netherlands Triage System
RE-AIM: Reach, Effectiveness and Efficacy, Adoption, Implementation, and Maintenance
SMASS: Swiss Medical Assessment System
SmED: Standardisierte medizinische Ersteinschätzung in Deutschland (*Standardized medical Initial Assessment in Germany* in English)
