# Supplementary material for: Implementation of a Standardized Initial Assessment for Demand Management in Outpatient Emergency Care in Germany: Early Qualitative Process Evaluation
Source: JMIR Form Res. 2020 Sep 4;4(9):e18456. doi: 10.2196/18456 (PMC7501577; doi:10.2196/18456)
Supplement: Multimedia Appendix 1 [file formative_v4i9e18456_app1.docx]

# Supplementary material

Translated interview guide used to conduct the qualitative interviews with healthcare professionals

| Implementation |
| --- |
| How was preparation for use and introduction of SmED organized? |
| Who was responsible for organizing preparation? |
| Are you aware of any strategies to ensure success of the preparation? |
| Regarding the preparation, what has been useful or unnecessary from your point of view? |
| What adjustments have been made prior to the implementation within your setting? |
| Prior to the implementation of SmED was there another form of initial assessment?  If yes, can you describe it? |
| Do you use an IT-System for practice management?  If yes, is the IT-System connected with SmED?  Could you describe how you document? |
| Effectiveness/Efficacy |
| Are you aware of situations were SmED ranked urgency differently compared to the system you used before or your own initial assessment?  If yes, what did you do? |
| Does SmED ranks patients higher compared to our own initial assessment? |
| Are you aware of any medical case were urgency is usually ranked higher by SmED compared to your own initial assessment? |
| Who has the medical responsibility within your setting regarding the use of SmED? |
| Can you describe the further procedure if a patient was ranked as non-urgent? |
| Are you aware of any changes regarding your work area which are due to implementation of SmED? |
| What factors influence the use of SmED? |
| Do you use the summaries SmED produces for each medical case? Do physicians read these summaries? |
| Adoption/Uptake |
| What expectations did you have regarding SmED?  Were your expectations met or were you disappointed? |
| What advantages or disadvantage do you seen in using SmED? |
| In your opinion, how does additional time requirement influence the further procedure (medical or organizational) of patients? |
| Sustainability/Maintenance |
| Which factors affect implementation sustainability? |
| What are the advantages and disadvantages of SmED if used at the joint counter in comparison to the use at the initial telephone contact point of the outpatient sector? |
| Do you have any suggestion for improvement? |
| Is there anything else you want to talk about? |
